# Repair of bilateral cleft lip and its variants

**DOI:** 10.4103/0970-0358.57194

**Published:** 2009-10

**Authors:** John B. Mulliken

**Affiliations:** Department of Plastic and Oral Surgery, Children's Hospital Boston and Harvard Medical School, Boston, Massachusetts

**Keywords:** Anthropometry, Bilateral cleft lip, Three-dimensional photogrammetry

## Abstract

The surgeon who lifts a scalpel to repair a bilateral cleft lip and nasal deformity is accountable for: 1) precise craftsmanship based on three-dimensional features and four-dimensional changes; 2) periodic assessment throughout the child's growth; and 3) technical modifications during primary closure based on knowledge gained from long-term follow-up evaluation. These children should not have to endure the stares prompted by nasolabial stigmata that result from outdated concepts and technical misadventures. The principles for repair of bilateral complete cleft lip have evolved to such a level that the child's appearance should be equivalent to, or surpass, that of a unilateral complete cleft lip. These same principles also apply to the repair of the variants of bilateral cleft lip, although strategies and execution differ slightly.

## INTRODUCTION

Surgeons seem resigned that a repaired bilateral cleft lip will be more noticeable than a repaired unilateral cleft lip. On the contrary, I have written that the appearance of a child with a repaired complete bilateral deformity should be comparable to, and in many instances, be better than that of a repaired unilateral complete cleft lip.[1] This optimistic statement is based on two major advances in the surgical management of bilateral cleft lip over the past quarter century. First is the realization for the need of preoperative manipulation of the protuberant premaxilla. Second is the acceptance of the principles of bilateral labial repair, especially the importance of simultaneous correction of the nasal deformity.

Preoperative dentofacial orthopedics is necessary to set the stage for synchronous, bilateral, nasolabial closure. Unfortunately, dentofacial orthopedics (active or passive) is often unavailable or considered unnecessary in many centers. Therefore, older techniques continue to be practiced: i) Staged repair: first, closure on one side of the lip (usually the more severely involved) and then, the other side. This often results in conspicuous nasolabial asymmetry. ii) There may be a role for preliminary labial adhesion, either bilateral or one side first; however, this method carries a risk of dehiscence. iii) The third common strategy is to undertake simultaneous bilateral labial repair over the protuberant premaxilla. This method makes it difficult to: 1) primarily correct the nasal deformity, and 2) design the philtral flap of appropriate dimensions in anticipation of growth. If healing is successful, the philtrum is likely to overgrow to become too wide or abnormally shaped like a shield or keystone. Children still present to cleft centers with their philtrum destroyed by geometric scars that result from misguided attempts to lengthen the prolabial element. There continues to be confusion about how to construct the median tubercle and prevent the familiar “whistling lip” deformity. Should the surgeon preserve the prolabial vermilion (resulting in a scar on each side), retain a tiny strip of the central vermilion, or excise it completely and build the tubercle using lateral labial elements (resulting in a central scar)?

It is the nasal shape rather than the look of the lip that brands the child with a repaired bilateral deformity. The short or “absent” columella seems to intimidate surgeons. Conventional wisdom has been to ignore the nasal distortion and focus on labial closure. There has been a lingering concern that dissection in the nose would compromise philtral circulation or interfere with nasal growth. Overlooked was the observation that primary labial closure accentuates the nasal deformation by pulling the medial crura to a more posterior position, further dislocating the alar domes and buckling the genua into recurvatum—the result is the “cat's knees” deformity.[2] The traditional solution was to address the nasal deformity when the child was older, employing a variety of techniques, often described as “columellar lengthening.” Unfortunately, these “secondary” procedures failed to result in a normal nasal appearance. Instead, they caused tertiary nasal distortions, each peculiar to the particular method used.[3]

### Principles

Surgical principles, once established, usually endure whereas surgical techniques continue to evolve. From a study of the literature and observations of residual deformities, the author induced the following principles for repair of bilateral cleft lip.[2]

- Maintain nasolabial symmetry because even the smallest differences become magnified with growth.
- Secure orbicularis oris continuity to construct the muscular ring and minimize philtral distortion.
- Design proper philtral size/shape because it rapidly elongates and widens (particularly superiorly).
- Construct the median tubercle using the lateral labial elements because retained prolabial vermilion lacks white roll and normal coloration and fails to grow to ample height.
- Position and secure the displaced lower lateral cartilages to establish normal nasal projection and columellar length.

Principles 1–4 needed confirmation, whereas principle 5 (synchronous repair of the nasal deformity) was a fundamental change in surgical strategy. It is unnecessary to drag up labial tissue to elongate the columella. “The columella is in the nose”—it can be exposed by anatomic positioning, apposition, and fixation of the lower lateral cartilages and sculpting the excess (expanded) skin in the nasal tip.[4]

## THIRD AND FOURTH DIMENSIONS

Like the sculptor working in marble, the surgeon must conceptualize the three-dimensional features during repair of the bilateral cleft lip and nasal deformity. Unlike sculpture in stone, however, the repaired lip and nose change with time. Thus, the surgeon must also understand the normal nasolabial alterations that occur with growth as well as the particular distortions that evolve in a child with repaired bilateral cleft lip and nasal deformity. Normal nasolabial growth patterns in Caucasians aged 1–18 years have been documented using anthropometry by Farkas *et al*.[5] A similar anthropometric study compared nasolabial dimensions in Indians to other ethnic groups[6] Fast-growing nasolabial features reach more than two-thirds of adult size by age five years. These include all dimensions except for columellar length (sn-c) and nasal tip protrusion (sn-prn). The stigmata of a repaired bilateral cleft lip correlate with these observations, *i.e*., fast-growing features become overly long or too wide, whereas slow-growing features remain short.[7] Therefore, features programmed for rapid growth in early childhood must be crafted on a small scale, whereas slow-growing features should be constructed on a slightly larger than normal size for an infant. The only exception to these guidelines is the formation of the median tubercle. This fast-growing feature lags behind in a repaired bilateral cleft lip, and therefore, must be made as full as possible.

## PREOPERATIVE DENTOFACIAL ORTHOPAEDICS

Alignment of the maxillary segments sets the stage for synchronous, bilateral, nasolabial repair. Retrusion and centralization of the premaxilla permits design of the philtral flap in proper proportions, facilitates nasal correction, and allows soft tissue closure of the alveolar clefts, which stabilizes the maxillary arch and eliminates oronasal fistulas. Furthermore, premaxillary retropositioning minimizes the nasolabial distortion that occurs during the rapid growth of early childhood.

There are two basic dentofacial orthopedic strategies: passive and active. A passive appliance maintains arch width, but requires some type of external force to retract the premaxilla. Bilateral labial adhesions have been tried for this purpose since the mid-19^th^ century; however, they often dehisce because of tension and absent muscle in the prolabium. Other methods include traction by an elastic band attached to a custom-fitted head cap or application of pressure on the prolabium by cheek-to-cheek tape. Cutting and Grayson have described a more sophisticated variation on a passive plate and taping called “nasoalveolar molding”(NAM).[8,9] After the alveolar gap is reduced, an acrylic extension is added to the palatal plate that pushes the nostrils upward against a counterforce of soft material across the nasolabial junction, thus stretching the diminutive columella. This apparatus must be re-taped and adjusted frequently, necessitating repeated visits over several months. Another type of passive plate and nasal outrigger, without any need for tape, has been reported by Bennun and Figueroa.[10]

The Latham appliance is the best-known active-type device for premaxillary moulding.[11] It is constructed from a plaster cast of the upper jaw. Most centers have the appliance fabricated in Ontario, Canada, although it can also be done locally. The device is pinned to the maxillary shelves, and the parents turn a ratcheted screw daily to expand the anterior palatal segments. Visits are necessary at one, three, and five weeks to tighten the bilateral elastic chain that retroclines the premaxilla. Usually, the premaxilla is aligned within 6–8 weeks.

The merits of active versus passive dentofacial orthopedic methods continue to be discussed by proponents of each approach. Cleft lip centers differ in their capability to provide this service. Dentofacial orthopedics may not be available in developing countries. Furthermore, infants in these nations often present when they are older than 6–12 months of age; by when, the premaxilla is rigid and manipulation is not possible. Another dilemma, sometimes seen in every land, is the twisted premaxilla that fails to be centralized despite several weeks of dentofacial manipulation. In these situations, the surgeon may resort to staged repair (with/without labial adhesion) or closure of the labial clefts over the procumbent premaxilla. Premaxillary ostectomy/setback and gingivoperiosteoplasties should also be considered in these predicaments.

Premaxillary retropositioning must be done with great care. Premaxillary circulation can be compromised by the mucosal incisions and dissection necessary for resection of the premaxillary neck and inferior septal cartilage, which is required to permit alveolar closure. With careful attention to mucosal blood supply, premaxillary retropositioning can be accomplished in an infant at the same time as nasolabial repair. If the child is near one year or older, then speech becomes the first priority. The strategy for these children is to first close the secondary palate and retroposition the premaxilla (with its labial blood supply), along with soft tissue closure of the alveolar clefts.[12] Nasolabial correction is scheduled later on a solid maxillary foundation. Primary premaxillary retropositioning might be called heretical because it is likely to accentuate midfacial retrusion, but the child's nasolabial appearance takes precedence. The majority of children with repaired bilateral complete cleft lip/palate will need maxillary advancement anyway, after completion of skeletal growth.[13]

## THE OPERATION

Bilateral cleft lip presents in three major anatomic forms: bilateral symmetrical complete (50%); bilateral incomplete (25%), and asymmetrical bilateral (complete/incomplete) (25%).[14] The technical steps are first described for the most common, the bilateral complete type, after which, modifications are suggested for the major variants.

*Note:* The day of bilateral cleft lip repair is the most important day in the child's life. It should be the first case of the morning. The surgeon must work slowly, carefully, and take as much time as necessary to do the very best operation. There should be no distractions such as scheduled meetings, patients waiting in the office, or other obligations for that day.

### Bilateral Complete Cleft Lip and Palate

#### Markings

**Figure 1:**
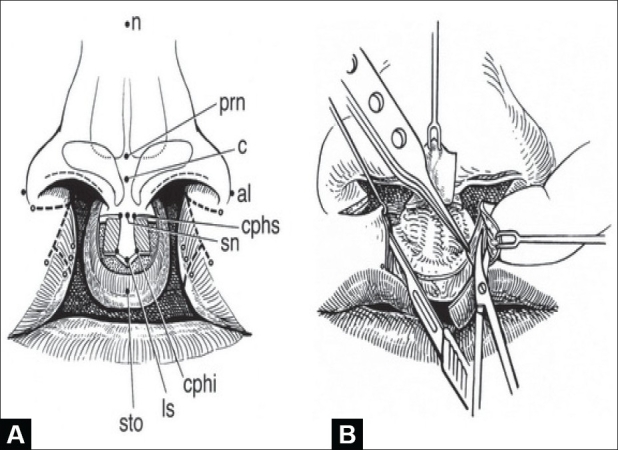
(A) Markings for synchronous repair of bilateral complete cleft lip and nasal deformity. Anthropometric points: n = nasion; prn = pronasale; al = alare; c = columella; sn = subnasale; cphs = crista philtri superior; cphi = crista philtri inferior; ls = labiale superius; sto = stomion. Open circles designate tattooed points, (B) Dissection of orbicularis oris muscle in lateral labial elements

The anatomic points are designated using standard anthropometric initialisms[15] [Figure 1A, left]. The philtral flap is drawn first while the nostrils are held upward with a double-ball retractor. A sharpened tooth-pick is used for drawing; brilliant green dye (tincture) is preferred over methylene blue (aqueous). The dimensions are determined by the age of the child and ethnicity. The average age at primary repair is five months. At this time, the length of the philtral flap is usually the same as the height of the cutaneous prolabium (usually 6–7 mm). If the prolabium is overly long, the philtral flap should be shortened to this length. Philtral flap width is set at 2 mm at the columellar-labial junction (cphs-cphs) and 3.5–4 mm between the proposed Cupid's bow peaks (cphi-chpi). The sides of the philtral flap should be drawn slightly concave because the scars tend to bow. The dart-like tip of the philtral flap should not be overemphasized. A thin rectangular flap is drawn on each side of the philtral flap. These side flaps will be de-epithelialized and will come to lie beneath the lateral labial flaps in an effort to simulate the elevation of philtral columns.[15]

The proposed Cupid's bow peaks are carefully sited on the lateral labial elements and marked just atop the white roll above the vermilion-cutaneous junction. These points are situated so that there is some medial extension of the white roll that will form the handle of the Cupid's bow and sufficient vermilion height to form the central raphe and median tubercle. Curvilinear incisions are drawn at the juncture of the alar bases and lateral labial elements. Nostril rim incisions are marked and extended along the inside edge of the upper columella. Nasal and labial segments are infiltrated with lidocaine (0.5%) / epinephrine (1:200,000). After waiting for five minutes, the critical points including the lateral vermilion-mucosal junctions, are tattooed with tincture of brilliant green [Figure 1A].

#### Labial Dissection

First, all labial incisions are lightly scored. The flaps flanking the philtral flap are de-epithelialized, the remaining prolabial skin is discarded, and the philtral flap is elevated (including subdermal soft tissue) off the premaxilla up to the anterior nasal spine. The white-roll-vermilion-mucosal flaps are incised (just short of the tattooed lateral Cupid's bow point) and the lateral labial elements are disjoined from the alar bases. These basilar flaps are freed from the piriform attachments by incision along the inferior cutaneous-mucosal (inter-cartilaginous) junction. The mucosal incisions on the underside of the lateral elements are extended distad, on the anterior side of the gingivolabial sulcus, to the premolar region. With a double-hook on the muscle layer, the lateral labial elements are widely dissected off the maxilla in the supraperiosteal plane. This permits greater advancement of the cheek than possible using subperiosteal dissection. A protective ring finger is held on the infra-orbital rim as this dissection extends over the malar eminence. Releasing the lip from the maxilla is a critical maneuver; this is needed to minimize tension on the muscular closure and permit tension-free cutaneous closure. The orbicularis oris bundles are dissected in both the subdermal and submucosal planes for 7–10 mm [Figure 1B].

#### Nasal dissection

**Figure 2:**
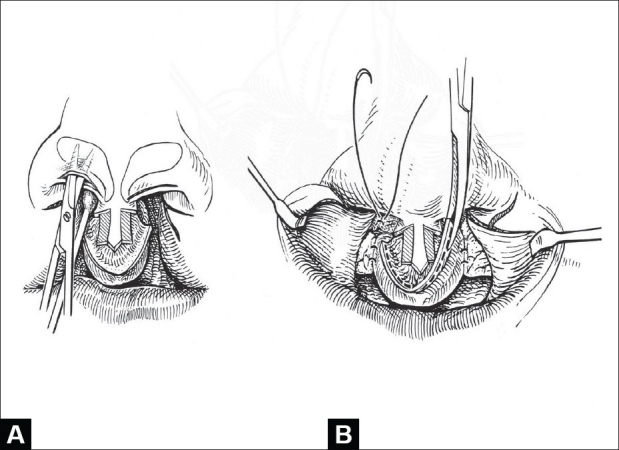
(A) Semi-open exposure of lower lateral cartilages through rim incisions. Cotton-tipped applicator held in upper vestibule helps display the splayed and dislocated cartilages, (B) Bilateral alveolar gingivoperiosteoplasties are complete and redundant premaxillary vermilionmucosa is being trimmed

Using a semi-open approach through bilateral rim incisions, the anterior surface of the slumped and splayed lower lateral cartilages is exposed by scissor-dissection, aided by elevation with a cotton-tipped applicator on the mucosal underside. This dissection continues superiorly over the upper lateral cartilages and across the dorsal septal junction [Figure 2A]. Interdomal fatty tissue is elevated and partially excised. Perichondrium-to-perichondrium heals more securely without intervening soft tissue.

#### Alveolar Closure

Vertical incisions are made on each side of the premaxilla and on the facing gingiva of the lesser segments. Alveolar gingivoperiosteoplasties are completed. The nasal floors are closed using a lateral mucosal flap raised from below the inferior turbinate and a medial flap from the premaxilla. The alar base flaps are advanced and sutured to the edge of the constructed nasal floor. The vermilion component of the premaxillary mucosa is trimmed and the remaining mucosal flange is secured high to the premaxillary periosteum to construct the posterior side of the central gingivolabial sulcus [Figure 2B].

#### Labial Closure

Advancement of the lateral labial elements during closure of the sulcus is difficult to illustrate. Nevertheless, this maneuver is critical to muscular closure, tension-free philtral closure, and a protrusive posture of the lip. A back-cut is made at the distal end of the sulcal incision, and the sulcus is closed while the labial flap is pulled mesially with a double hook. The lateral labial mucosal lining forms the anterior wall of the central gingivolabial sulcus.

**Figure 3:**
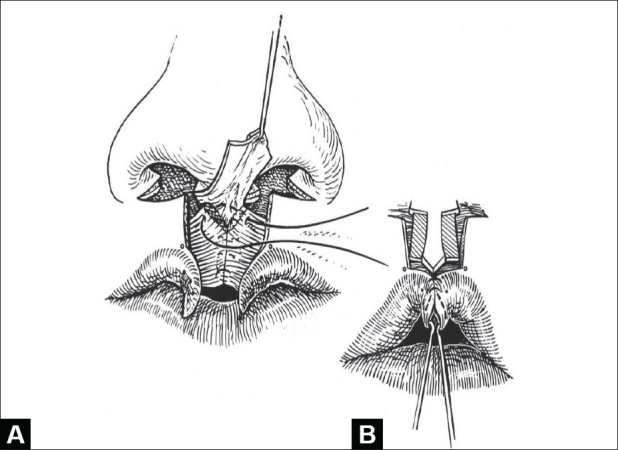
(A) Apposition of orbicular muscle bundles; upper suture attaches nasalis muscle to periosteum of anterior nasal spine, (B) Lateral white roll-vermilion-mucosal flaps used to construct Cupid's bow and full median tubercle. Redundant vermilion-mucosa trimmed to form median raphe

The orbicular bundles are apposed, end-to-end (inferiorly-to-superiorly), using either a vertical mattress or simple polydioxanone sutures. Polypropylene suture is used to suspend the pars peripheralis and nasalis to the periosteum of the anterior nasal spine [Figure 3A].

Construction of the median tubercle begins with the insertion of a fine chromic suture to join the white-roll-vermilion flaps at the midline; this is placed about 3 mm medially from the tattooed lateral Cupid's bow point [Figure 3B]. Excess vermilion-mucosa is trimmed from each flap and the junction aligned to form the median raphe. There is a natural inclination to save too much vermilion-mucosa in these flaps resulting in a central furrow.

Attention is turned to correction of the nose before insetting the philtral flap.

#### Nasal Correction

**Figure 4:**
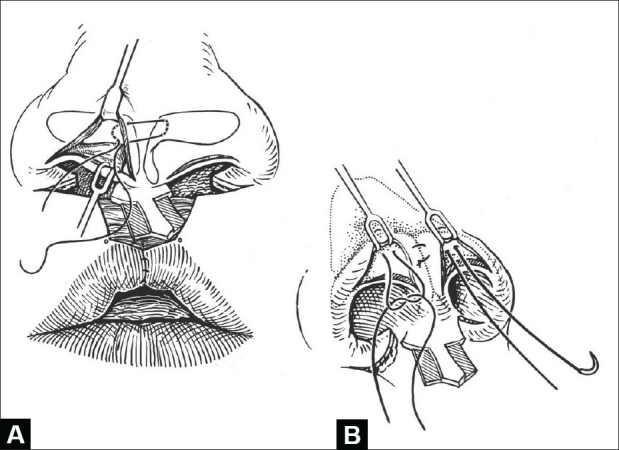
(A) Placement of interdomal mattress suture, (B) Insertion of mattress sutures to suspend genua and lateral crura over upper lateral cartilages

Three techniques have been described to suspend and secure the displaced lower lateral cartilages: 1) bolster sutures; 2) transfixion sutures, and 3) intercartilagineous sutures. The first two suture methods are done blindly; the third semi-open approach is preferred. Under vision, the genua are apposed with a 5-0 polydioxanone (1/2 circle cutting needle) [Figure 4A]. The lateral crura are elevated and secured to the ipsilateral upper lateral cartilage with a 5-0 polydioxanone mattress suture; a cotton-tipped applicator in the nostril beneath the genu facilitates inserting and tying these sutures [Figure 4B]. In an infant, usually only one upper-to-lower lateral suture is necessary (or possible), whereas 2–3 such suspension sutures are placed in an older child.

**Figure 5:**
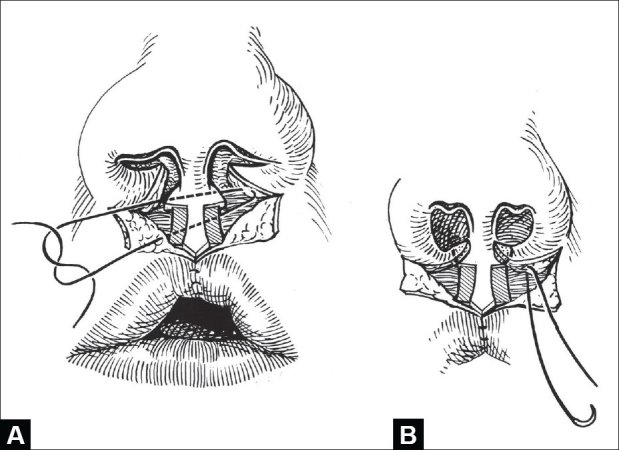
(A) Cinch suture to narrow interalar width, left untied until inset of alar base flaps, (B) Alar base flaps sutured side-to-end to columellar c-flaps and trimmed After sill is closed, suture placed earlier in maxillary periosteum is brought through alar base and tied to secure base and configure lateral sill

The c-flap on each side of the columellar base is trimmed to 3 mm in length. The alar bases are advanced, rotated endonasally, and sutured side-to-end to the c-flaps. Usually, the tip of the alar base flap is conservatively trimmed as closure of the sill is completed. A polydioxanone suture is placed through the maxillary periosteum in the region of the depressor alae nasi and left untied. This is best done prior to apposition of the upper orbicularis oris. A “cinch” suture of polypropylene is brought through the dermis of each alar base, passed beneath the philtral flap, and tied to narrow the interalar distance (al-al) to less than 25 mm [Figure 5A]. The previously placed maxillary periosteal suture is brought above the pars peripheralis and nasalis, then though the alar base, and tied. This suture simulates the depressor alae nasi and: 1) gives a cymal shape to the sill; 2) prevents alar elevation with smiling; and 3) minimizes postoperative nasal widening [Figure 5B].

#### Final Touches

In an effort to form the dimple, a suture is brought through the dermis in the lower one-third of the philtral flap and through the underlying orbicular muscular layer. The tip of the philtral flap is inset into the handle of Cupid's bow. In a complete bilateral cleft lip, it is unnecessary to adjust the leading edge of the lateral labial flaps before apposing them to the philtral flap with interrupted, fine dermal and percutaneous sutures. The cephalic margin of the labial flaps must be trimmed to correspond to the position and cymal configuration of the alar bases. Labial flap-to-sill closure proceeds laterally-to-medially.

**Figure 6:**
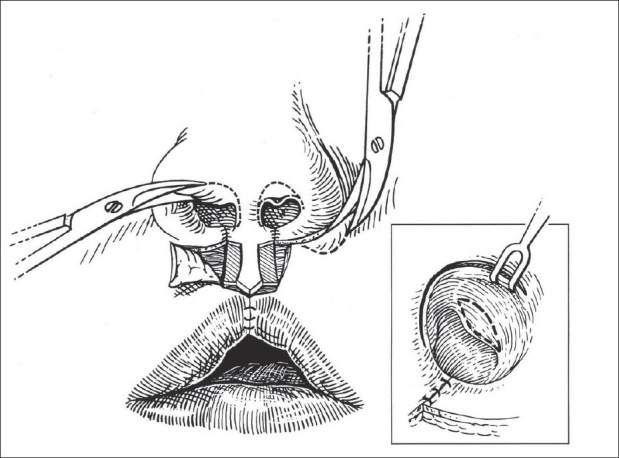
Cymal resection of upper edge of lateral labial flap and crescentic resection of redundant skin in soft triangle and upper columella. Inset shows lenticular excision of extra vestibular lining to correct lateral web

After anatomic placement of the lower lateral cartilages, it is obvious that there is redundant domal skin in the soft triangles and upper columella. A crescentic excision of this extra skin is drawn along the leading edge of the rim incisions, extending inferiorly along each side of the columella [Figure 6]. This resection narrows the nasal tip, defines and tapers the mid-columella, and elongates the nostrils. Interdomal apposition also accentuates the extra lining in the lateral vestibule; this is corrected by lenticular excision [Figure 6, inset].

**Figure 7:**
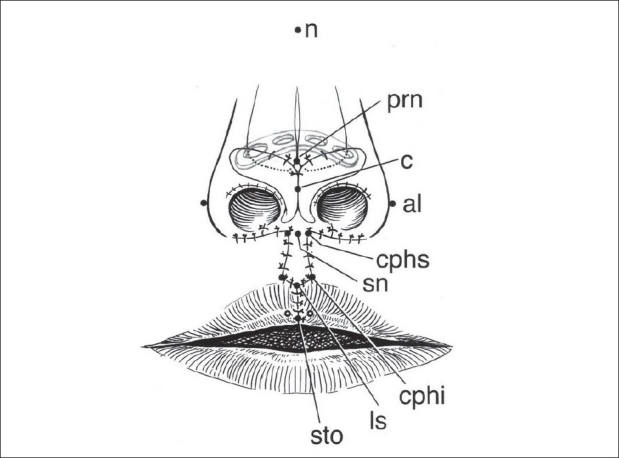
Completed repair. Anthropometric measurements document baseline nasolabial dimensions

An internal resorbable nasal splint is used rather than an external splint. A short, curved polylactic-polyglycolic resorbable plate is inserted into the nasal pocket above the newly positioned lower lateral cartilages.[16] Immediate postoperative nasolabial anthropometry is documented and placed in the child's record[15] [Figure 7]. After measuring, a strip of 1/4 inch Xeroform^®^ gauze is wrapped around a 19 gauge silicone catheter and a 1 cm segment is inserted into each nostril.

#### Postoperative Care

**Figure 8:**
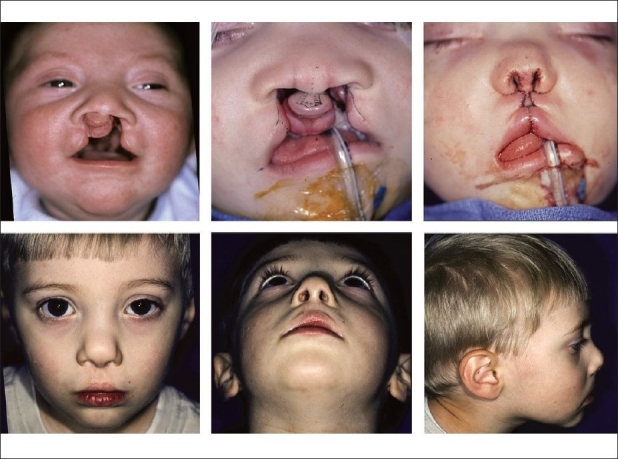
(A) Preoperative image of newborn with bilateral complete cleft lip/palate prior to dentofacial orthopaedic manipulation, (B) Intraoperative markings: note philtral flap designed 2.0 mm at cphs-cphs and 4.5 mm at cphi-cphi.with lateral tabs to be de-epithelialized, (C) Following completion of nasolabial repair; postoperative anthropometric dimensions are recorded, (A, B & C) Appearance at age 2.5 years

A Logan bow is taped to the cheeks to: 1) protect the labial repair and 2) hold an iced-saline sponge for 24 hours postoperatively. The infant is usually discharged from hospital on postoperative day #2. The parents are instructed in suture-line care. The percutaneous sutures are removed and the nostrils are cleaned 5–6 days postoperatively under general anesthesia using mask induction and insufflation. A ½ inch transverse Steri-Strip™ (3M Health Care, St. Paul, Minnesota) is trimmed to fit the sn-ls dimension and placed over the labial scar; this tape is changed as needed for six weeks. Thereafter, parents are instructed on how to perform digital massage and warned about the importance of the application of a sunblock ointment [Figure 8].

## BILATERAL CLEFT LIP VARIATIONS

### Binderoid bilateral complete cleft lip and palate

The nasal features in this rare variant are: orbital hypotelorism, hypoplastic bony/cartilaginous elements, conical columella, short septum, and absent anterior nasal spine. The labial features are: hypoplastic prolabial-premaxillary segment (containing a single tooth bud) and thin vermilion in the lateral labial elements.[17] Dentofacial orthopedic manipulation is usually not possible because the premaxilla is floppy; furthermore, it is also unnecessary because the premaxilla is not protuberant. Synchronous repair is accomplished as described above. Sometimes, the premaxilla is so small that alveolar gingivoperiosteoplasties cannot be accomplished. The philtral flap need not be made overly small because it will expand very little with growth (because the premaxilla is so tiny). The lower lateral cartilages are very small; however, they can usually be dissected, positioned, apposed, and elevated.

Specific secondary procedures in this variant include dermal grafting to augment the median tubercle and to widen the narrow columellar base; possible cartilage grafting of the tip and nasal dorsum; and maxillary advancement along with augmentation of the *fossae praenasale.*[17]

### Bilateral complete cleft lip and intact secondary palate

Approximately 10% of infants born with bilateral complete cleft lip and cleft alveolus have an intact secondary palate. The premaxilla is procumbent and unyielding to any attempts at dentofacial orthopedic manipulation. This situation is another indication for premaxillary set-back undertaken synchronously with nasolabial repair. There are two alternatives to ensure adequate premaxillary blood supply through the mucosa/periosteum: 1) postpone attachment of the posterior edge of the premaxilla to the anterior edge of the hard palate, or 2) delay alveolar gingivoperiosteoplasties. Midfacial retrusion is very unlikely to evolve in the presence of an intact secondary palate.

### Bilateral incomplete cleft lip

**Figure 9:**
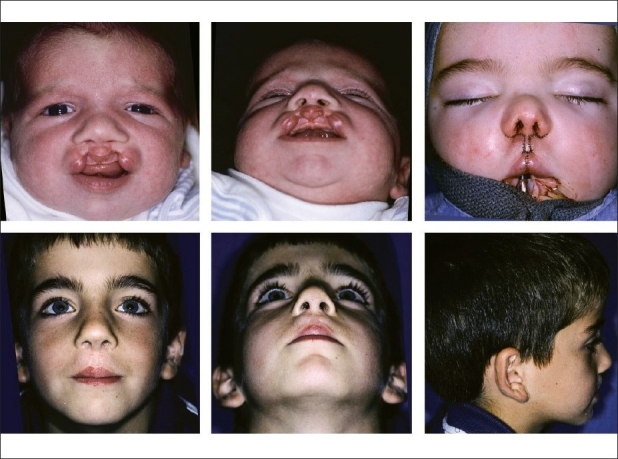
(A). Infant with bilateral symmetrical incomplete cleft lip and intact secondary palate, (B) Submental view, (C) Following synchronous nasolabial repair, including positioning and fixation lower lateral cartilages, (A, B & C) Appearance at age six years

One-fourth of bilateral cleft lips are bilaterally incomplete; most are symmetrical.[14] This variant is the most easily corrected of all. The concepts for design and execution are the same as for a complete bilateral cleft lip, including the need for adjustments based on expected nasolabial changes with growth. Nevertheless, there are crucial technical points that must be considered. The first relates to the construction of the median tubercle. Usually, the tubercle should be formed using the lateral white roll-vermilion-mucosal flaps. However, if the clefts are minor (< 50% of the cutaneous lip) and the central white-roll is prominent, the prolabial vermilion-mucosal may be retained as the central segment with flanking scars. The next consideration is the columella: measure its height (sn-c). If the columellar length is normal and the lower lateral cartilages are in reasonable position, then it may be unnecessary to adjust the lower lateral cartilages and sculpt the nasal tip. Interalar narrowing is always needed as this dimension is overly wide preoperatively and increases with growth. If there is any separation of the alar domes, they should be apposed through the semi-open approach. In contrast to the complete deformity, both the leading and superior edges of the lateral labial flaps may have to be trimmed [Figure 9].

### Asymmetrical bilateral (complete/incomplete) cleft lip

One-fourth of bilateral cleft lips are asymmetrical: complete on one side and incomplete on the other side.[14] Severity of the cleft on the incomplete side determines the operative strategy. The following classification system of incomplete clefting is useful in categorizing the types of repair for these asymmetrical variants.

“Incomplete” is a general term applied to a cleft lip in which there is cutaneous continuity between the medial (nasomedial process) and lateral (maxillary process) elements. Incomplete cleft lips present in a wide spectrum, beginning at the severe end with those with a thin cutaneous band (that some would argue constitutes a “complete” cleft lip) to lesser-forms that have been called by various names, *e.g*., microform, minimal, and occult. Yurzuriha and Mulliken subdivided these lesser-forms into *minor-form*, *microform*, and *mini-microform* as determined by the degree of disruption at the vermilion-cutaneous junction.[18] Minor-form cleft lip extends 3–5 mm above the normal Cupid's bow peak, *i.e*., 50% or less of the normal cutaneous labial height (sbal-cphi). Microform cleft lip is characterized by a notched vermilion-cutaneous junction, whereas the Cupid's bow peak is elevated < 3 mm above normal. The mini-microform cleft lip is distinguished by a discontinuous white-roll without elevation of the Cupid's bow peak. The severity of the nasal deformity, muscular depression, and mucosal notching correspond in these three categories of lesser-form cleft lip.

**Figure 10:**
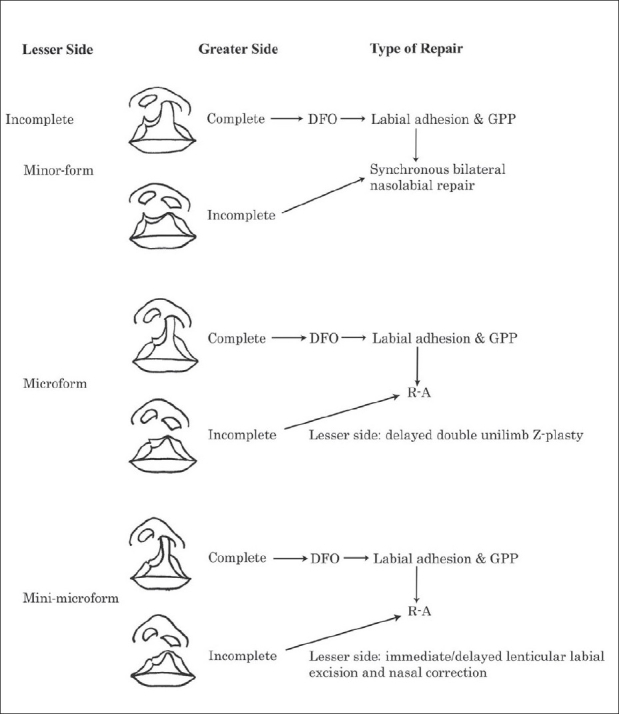
Algorithm for repair of bilateral asymmetrical cleft lip (complete or incomplete) and contralateral incomplete or lesser-form cleft (Modified from Yuzuriha, Oh, Mulliken, 2008)

**Figure 11:**
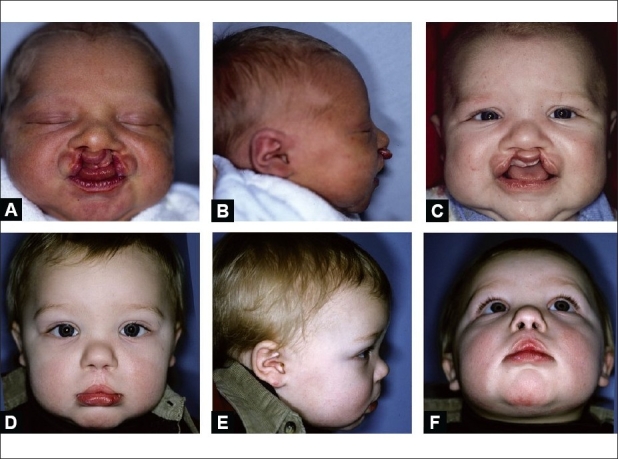
(A) Bilateral asymmetrical cleft lip: complete on right and incomplete on left, (B) Lateral view, (C) Following left labial adhesion, (D, E & F) Age one year after second stage bilateral nasolabial repair

Symmetry, the first principle of bilateral labial repair, is the foremost concern in planning correction of these asymmetrical variants. An algorithm for timing and techniques for repair of asymmetrical bilateral cleft lip is shown in Figure 10.[14] If both greater and lesser sides are incomplete clefts or the lesser side is minor-form, then synchronous bilateral repair is indicated. However, if the greater side is incomplete, it alone is first repaired if the contralateral side is a microform or mini-microform. If the greater side is complete, it is initially addressed by unilateral dentofacial orthopedics and followed by nasolabial adhesion and alveolar gingivoperiosteoplasty; this levels the surgical field. If the contralateral (lesser side) cleft lip is a minor-form or a more severe incomplete form, the next procedure is simultaneous bilateral nasolabial repair. During this second stage, technical maneuvers on the complete side are exaggerated because the distortions and tensions are greater than on the incomplete side [Figure 11]. If the contralateral cleft is a microform, it is best to close only the complete side, following the rotation-advancement principle, along with primary nasal repair. After the scar has remodeled, the contralateral microform is repaired using a double unilimb z-plasty, which includes muscular reapposition, dermal graft, and nasal correction.[19] This three-stage stratagem is most likely to result in acceptable mirror-image nasolabial symmetry.

**Figure 12:**
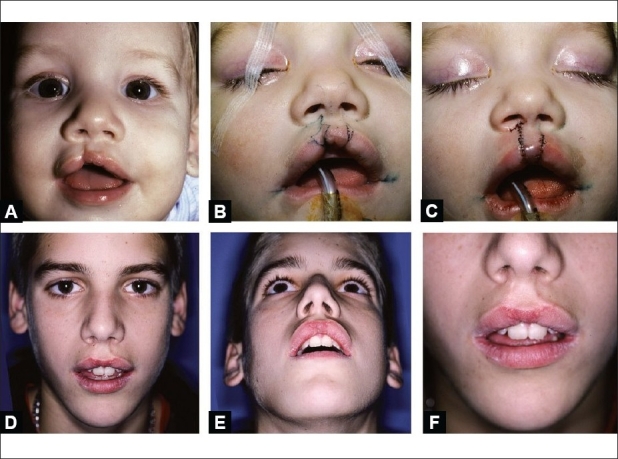
(A) Infant with bilateral asymmetric cleft lip: minor-form on right and mini-microform on left, (B) Intraoperative markings for right abridged rotation-advancement repair and left vertical lenticular excison of the minimicroform, (C) Photograph following correction, (D, E & F) Age 15 years. Note: slightly wide cphi-cphi (necessary to match cphi sites), mismatched color and weakness of median tubercle, and furrow lateral to left philtral column

If the contralateral cleft lip is a mini-microform, this can be addressed along with repair on the greater side, although nothing is lost by waiting [Figure 12]. Often, only minor adjustment of the nasal tip is needed if there is a contralateral mini-microform. Augmentation of the median tubercle is almost always necessary.[14]

## PERIODIC ASSESSMENT

The surgeon's responsibility does not end with completion of the nasolabial repair. There is an obligation to periodically assess the outcome of the procedure. This is essential so that the surgeon will come to understand the changes in the fourth dimension, learn from these observations, and apply this knowledge to the correction of the next child who presents with a bilateral cleft lip.

### Photography

Preoperative photographs are a basic minimum for documentation. The surgeon must be patient in taking preoperative frontal, submental, and lateral views of the infant. Intraoperative photographs should be taken after cutaneous markings and immediately after repair. The standard angle of the submental view aligns the tip of the nose on a line sighted half-way between the medial canthi and eyebrows. The submental photograph is essential for assessment of nasal symmetry and configuration. Photographs must also be taken periodically during childhood, as well as prior to and after the adolescent growth spurt.

### Revision-Rate

Most surgeons keep a mental tally of the kinds of revisions needed after cleft nasolabial repair. Knowledge of the types of secondary corrections guides the surgeon's technical alterations during subsequent primary procedures. The goal is to minimize revisions. The cutaneous lip should never need reopening and the nasal cartilages rarely require repositioning, whereas readjustment of the mucosal free margin and nasal width are commonly necessary.

Nasolabial asymmetry, distortion, and hypoplasia become increasingly obvious prior to attending school. These problems usually remain relatively unchanged throughout childhood, only to become magnified during adolescence. School age is the first time to make a formal assessment of outcome based on types of revision. In a study of 50 consecutive nonsyndromic children (median age 5.4 years), the revision rate was 33% for bilateral complete lip/palate and 12% for bilateral complete lip/alveolus with an intact secondary palate.[20] Resuspension of prolapsed anterior gingivolabial mucosa was the most frequent labial revision. Dermal graft augmentation of the median tubercle was commonly done at the time of alveolar bone grafting (age 9–11 years). The most frequent nasal distortion was disproportionate widening of the interalar dimension, although this rarely required correction during childhood. None of the children required a secondary “columellar lengthening” procedure. Reassessment of frequency and types of nasolabial revisions must be undertaken after completion of skeletal growth.

Revision-rate is only a qualitative measure of outcome, *i.e*., determined by the surgeon's subjective assessment, often made in agreement with the family and patient. Quantitative methodology is necessary, which can be used for interinstitutional comparative studies. Cephalometry has long been used by dental specialists to document skeletal growth in children with repaired cleft lip/palate. A similar method is needed to accurately and simply measure nasolabial appearance.

### Direct anthropometry

Farkas was the first to apply medical anthropometry to children with repaired bilateral cleft lip.[21] Normative values for nasolabial features for Caucasian children from infancy through adolescence are tabulated in his publication[5] and in his book.[22] Direct anthropometry requires training and practice; the tools are a sliding Vernier caliper and a Castroviejo caliper. Anthropometry can be used intraoperatively to document the severity of the deformity and to record baseline nasolabial dimensions immediately following repair.[15] Direct anthropometry is repeated as the child grows and compared to normal values by sex and age.[4] Caution: locating the soft tissue landmarks and measuring nasolabial dimensions is difficult in a child who is less than age five years, although it is quite easily done in an older child.

### Indirect anthropometry

Photogrammetry (a type of indirect anthropometry) eliminates the inaccuracies of measuring the lip and nose in a frightened or fidgety child. However, measuring a photograph introduces errors due to magnification, variation in lightening, head orientation, and subject-to-camera distance. The magnification factor can be eliminated by including a standard metric in the photograph. Even so, photogrammetry is two-dimensional; it can only be used to measure certain nasolabial features, proportions, and angles.[23]

**Figure 13:**
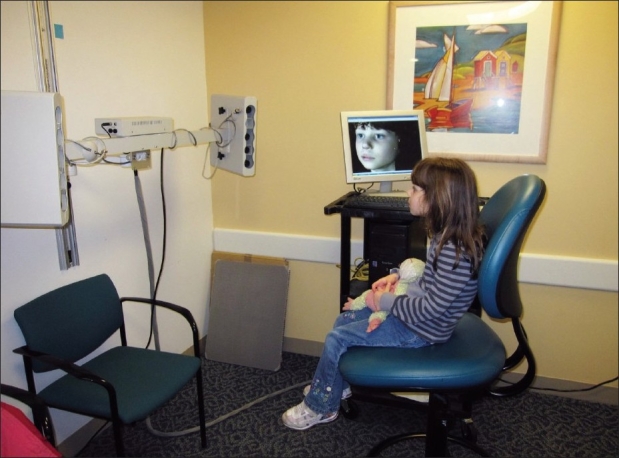
Young patient sitting in front of 3D photogrammetric cameras

**Figure 14:**
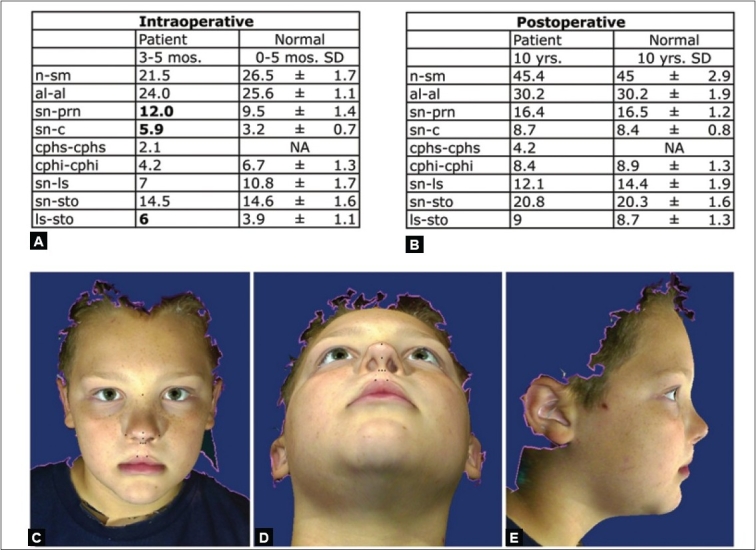
(A) Direct anthropometry of patient in Figure 11 compared to normal age- and sex-matched values, (B) 3D photogrammetric indirect anthropometric dimensions of patient in Figure 11 at age ten years compared to age-and sex-matched normal values, (C, D & E) 3D photogrammetric computer images of patient in Figure 11 at age ten years: dots placed to determine nasolabial dimensions

**Figure 15:**
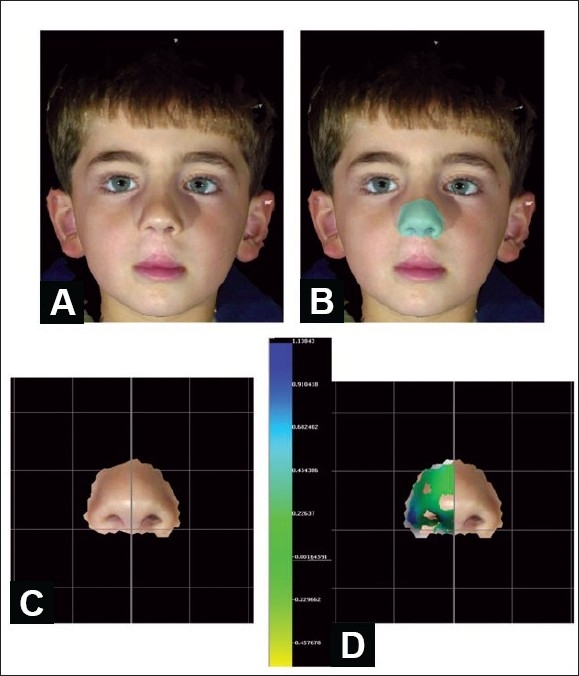
Example of 3D photogrammetric measurement of nasal symmetry (A) 3D image of five year-old boy with repaired bilateral incomplete cleft lip (Also in Figure 9), (B) Nose highlighted, (C) Nasal 3D image divided at midplane, (D) Mirror image of left heminose superimposed on right heminose. Canfield Vectra 3D software analysis: mean difference in projection between pixels on the two sides of the nose is 0.19 ± 0.3 mm. Color bar indicates topography: Blue = left heminose more projected; Green= same projection; Defect = left heminose less projected than natural-colored right heminose

Three-dimensional stereo-photogrammetric systems are now available: 3dMDface (3dMD, Atlanta, GA) and Vectra 3D (Canfield Imaging Systems, Fairfield, N.J.). Synchronized high-resolution digital cameras capture images in milliseconds [Figure 13]. Software algorithms merge the different overlapping images into a single three-dimensional image that can be viewed, turned in any direction, and analyzed on any computer. The validity and reliability of these systems has been documented.[24,25] Standard anthropometric points can be easily located after the image is appropriately maneuvered and nasolabial dimensions are recorded [Figure 14]. The digital images can be manipulated to calculate the soft tissue projection and to produce a numeric value for the overall mirror-image nasolabial symmetry [Figure 15].

## CONCLUSIONS

A child born with bilateral cleft lip should not have to suffer because of an ill-conceived and poorly executed primary repair. The operative principles for synchronous nasolabial correction are established. The techniques are within the repertoire of any well-trained and careful surgeon who is genuinely focused on the care of these children. Philtral columns and dimple are the only labial features that seem to be just beyond the surgeon's craft.

For too long, there has been misplaced emphasis on protocols that are based on the traditional concept of trying to minimize inhibition of midfacial growth. Indeed, some degree of maxillary retrusion is an unavoidable consequence of closure of the lip and palate. First priorities are nasolabial appearance and speech. Midfacial hypoplasia and underbite are entirely correctable after completion of facial growth.

Some type of dentofacial orthopedic manipulation is necessary to permit proper philtral design, nasal correction, and alveolar closure. If dentofacial orthopedics fails or if the child presents too late for an appliance to work, consider premaxillary ostectomy and set-back, either at the time of nasolabial repair (in infancy) or synchronously with palatal closure (in late infancy or early childhood).

The surgeon must correct the bilateral lip and nasal deformity in three-dimensions while making alterations based on anticipated changes in the fourth-dimension. This understanding is only gained by documenting the changing nasolabial features in children with repaired bilateral cleft lip and nasal deformity. Photography is essential and tabulation of revision-rate is useful; however, the future is in computerized three-dimensional photogrammetry.
